# Resveratrol alleviates lipopolysaccharide-induced liver injury by inducing SIRT1/P62-mediated mitophagy in gibel carp (*Carassius gibelio*)

**DOI:** 10.3389/fimmu.2023.1177140

**Published:** 2023-04-24

**Authors:** Liyun Wu, Qiaozhen Chen, Bo Dong, Hancheng Geng, Yu Wang, Dong Han, Xiaoming Zhu, Haokun Liu, Zhimin Zhang, Yunxia Yang, Shouqi Xie, Junyan Jin

**Affiliations:** ^1^ State Key Laboratory of Freshwater Ecology and Biotechnology, Institute of Hydrobiology, Chinese Academy of Sciences, Wuhan, China; ^2^ College of Advanced Agricultural Sciences, University of Chinese Academy of Sciences, Beijing, China; ^3^ The Innovative Academy of Seed Design, Chinese Academy of Sciences, Beijing, China

**Keywords:** resveratrol, lipopolysaccharide, oxidative stress, inflammation, mitophagy

## Abstract

**Introduction:**

Resveratrol (RES) is a polyphenol organic compound with antioxidant and anti-inflammatory properties. This study aimed to determine whether and how RES can alleviate liver injury in lipopolysaccharide (LPS)-induced gibel carp.

**Methods:**

Gibel carp were fed a diet with or without RES and were cultured for 8 weeks, followed by LPS injection.

**Results and discussion:**

The results suggested that RES attenuated the resulting oxidative stress and inflammation by activating the Nrf2/Keap1 pathway and inhibiting the NF-κB pathway, as confirmed by changes in oxidative stress, inflammation-related gene expression, and antioxidant enzyme activity. Furthermore, RES cleared damaged mitochondria and enhanced mitochondrial biogenesis to mitigate reactive oxygen species (ROS) accumulation by upregulating the SIRT1/PGC-1α and PINK1/Parkin pathways and reducing p62 expression. Overall, RES alleviated LPS-induced oxidative stress and inflammation in gibel carp through mitochondria-related mechanisms.

## Graphical Abstract

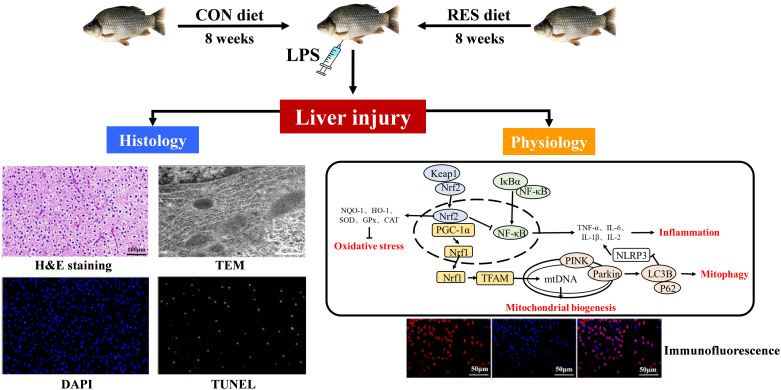

## 1 Introduction

Aquaculture makes a major contribution to human nutrition by providing a large proportion of high-quality animal protein for consumption (1). Thus, the safety of aquatic products is vital for human wellbeing. However, the deterioration of the aquaculture environment can cause oxidative stress, inflammatory reactions, and tissue damage in fish, then affecting the safety of aquatic products (2). Lipopolysaccharide (LPS), a main component of the outer membrane of Gram-negative bacteria, is composed of lipids and polysaccharides, has been found to be responsible for the pathogenicity of several bacterial diseases in fish (3). As in mammals, LPS injection in fish induces nuclear factor-kappa B (NF-κB) to disengage from inhibitor of NF-κB alpha (IκBα) and to be translocated to the nucleus, where it enhances the expression of proinflammatory cytokines including interleukin 1β (IL-1β) and tumor necrosis factor alpha (TNF-α), consequently leading to inflammation (4). Concurrently, a burst of reactive oxygen species (ROS) breaks the balance with the antioxidant defense system and impels the nuclear factor erythroid 2-related factor (Nrf2) that was originally bound to Kelch-like ECH-related protein 1 (Keap1) to dissociate and bind to the antioxidant response element (ARE) in the nucleus (5). Then, transcripts of downstream genes including heme oxygenase-1 (HO-1), NAD(P)H: quinone oxidoreductase 1 (NQO1), glutathione S-transferase (GST), and glutathione peroxidase (GPx) are activated (6, 7). Similarly, it has been reported that LPS induces oxidative stress, inflammation, and apoptosis in zebrafish (8), carp (9), and yellow catfish (10).

Mitochondria act as the main site of ROS generation, and they possess a variety of quality control mechanisms to maintain their functioning and homeostasis. Evidence has shown that after LPS injection, silent information regulator 1 (SIRT1) relies on NAD^+^ to mediate the deacetylation of Nrf2 and then specifically binds to the ARE located in the p62 promoter (11). P62 and oxidized proteins are transmitted to autophagosomes for degradation, and thus, the balance of ROS in the animal can be maintained (12). However, mitochondrial dysfunction may occur when excessive ROS exist *via* triggering the forkhead box protein O3 (FOXO3) signaling pathway and upregulating the expression of autophagy-related genes such as microtubule-associated protein 1A/1B-light chain 3 (LC3). The autophagy core protein LC3 is converted from LC3 I to LC3 II when autophagy is activated, and the latter acts as a structural protein of autophagosomes associated with the clearance of damaged mitochondria (13, 14). Normally, mitophagy is a protective mechanism against mild and moderate stress. However, over-activated mitophagy could induce cell death. For mitochondrial homeostasis, SIRT1 mediates the activation of nuclear respiratory factors 1 and 2 (NRF1 and NRF2) by peroxisome proliferator-activated receptor (PPAR) γ co-activator 1α (PGC-1α) and mitochondrial transcription factor A (TFAM) to induce mitochondrial biogenesis (15–17). Subsequently, mtDNA increases and ROS production decreases. However, the occurrence of LPS-induced mitophagy and mitochondrial biogenesis in fish has not been fully described.

At present, Chinese herbs are being increasingly used to prevent and cure diseases in aquaculture species by promoting immune function (18, 19). Resveratrol (RES), as a typical activator of SIRT1, is a non-flavonoid polyphenol organic compound derived from red grapes and other foods that can reduce the ROS and mitochondrial superoxide production caused by high levels of glucose (20). RES can decrease lipid oxidative damage in southern flounder (*Paralichthys lethostigma*) (21). In turbot (*Scophthalmus maximus*), RES alleviates oxidative stress and inflammatory reactions induced by soybean meal (22). Similarly, RES defenses liver damage and metabolic imbalances in common carp (*Cyprinus carpio*) (23). Thus, RES can reduce oxidative damage and enhance immunity in fish. In addition, RES can promote mitophagy and attenuate oxidative stress-induced tissue damage by regulating mitochondrial redox homeostasis and function *via* the SIRT1/PGC-1α signaling pathway (24). Gibel carp (*Carassius gibelio*) is an economically important freshwater fish in China, with the qualities of rapid growth and strong disease resistance. In the previous study, we found that RES could attenuate oxidative stress, inflammation, and mitochondrial dysfunction in gibel carp under acute ammonia exposure (25). Thus, we hypothesized that RES could attenuate LPS-induced liver injury in gibel carp by alleviating oxidative stress and the inflammatory reaction and by regulating mitophagy and mitochondrial biogenesis. To verify our hypothesis, fish were fed a diet with or without RES and were cultured for 8 weeks and then subjected to LPS injection. The biochemical indexes, histological changes, and gene and protein expression levels of the fish were then analyzed. Our study elucidates effects of RES on oxidative stress and inflammation in LPS-induced gibel carp for the first time, and provides an effective strategy to mitigate LPS-induced liver damage in fish.

## 2 Materials and methods

### 2.1 Experimental animals

Gibel carp were from the Institute of Hydrobiology, Chinese Academy of Sciences (Wuhan, Hubei, China). The experimental procedures on animals were approved by the ethics committee of the Institute of Hydrobiology, Chinese Academy of Sciences (Approval ID: IHB20140724).

### 2.2 Experimental protocol

Two isonitrogenous (36% crude protein) isoenergetic (7% crude lipid) diets supplemented with or without 500 mg/kg RES (≥ 98%; Solarbio, Beijing, China) were formulated for the experiment as described previously (25). The diet ingredients and chemical composition are shown in **Table 1**. Before the formal experiment, fish were temporarily raised in a circular aquaculture system for acclimation. Then, 180 healthy individuals initially weighing an average of 6.66 ± 0.01g were randomly assigned among six tanks (150 L water volume, 67 cm × 52 cm × 46 cm) and fed with a CON or RES diet three times per day (8:30, 13:30, and 18:30) for 8 weeks. The water temperature was maintained at 29–31°C; total ammonia nitrogen was < 0.1 mg/L; the dissolved oxygen was > 7.0 mg/L, and the photoperiod was 12L:12D.

**Table 1:** Formulation and approximate composition (% dry matter) of practical diets supplemented with resveratrol (RES).

Ingredients	CON	RES
White fish meal^1^	15	15
Rapeseed meal^2^	20	20
Soybean meal^2^	25	25
Wheat flour	25.4	25.4
Oil mixture^3^	5.5	5.5
RES	0	0.016
Vitamin premix^4^	0.39	0.39
Choline chloride	0.11	0.11
Mineral premix^5^	5	5
Carboxy methyl cellulose sodium	3	3
Cellulose	0.60	0.58
Proximate chemical composition (%)		
Crude protein (%)	36.83	36.32
Crude lipid (%)	6.94	6.89
Moisture (%)	9.90	9.12
Ash (%)	6.58	6.92

^1^ White fish meal: Purchased from American Seafood Company, Seattle, Washington, USA.

^2^ Soybean and rapeseed meal: Purchased from Coland Feed Co. Ltd., Wuhan, Hubei, China.

^3^ Oil mixture: soybean oil: fish oil = 1:1.

^4^ Vitamin premix (mg·kg^−1^ diet): Vitamin B_1_, 20; Vitamin B_2_, 20; Vitamin B_6_, 20; Vitamin B_12_, 0.02; folic acid, 5; calcium pantothenate, 50; inositol, 100; niacin, 100; biotin, 0.1; cellulose, 3522; Vitamin C, 100; Vitamin A, 110; Vitamin D, 20; Vitamin E, 50; Vitamin K, 10.

^5^ Mineral salt premix (mg·kg^−1^ diet): NaCl, 500.0; MgSO_4_·7H_2_O, 8155.6; NaH_2_PO_4_·2H_2_O, 12500.0; KH_2_PO_4_, 16000; Ca(H_2_PO_4_)·2H_2_O, 7650.6; FeSO_4_·7H_2_O, 2286.2; C_6 h10_CaO_6_·5H_2_O, 1750.0; ZnSO_4_·7H_2_O, 178.0; MnSO_4_·H_2_O, 61.4; CuSO_4_·5H_2_O, 15.5; CoSO_4_·7H_2_O, 0.91; KI, 1.5; Na_2_SeO_3_, 0.60; Corn starch, 899.7.

After the feeding experiment, gibel carp were fasted for 24 h. The fish were lightly anaesthetized with MS-222 at a concentration of 60 mg/L and then weighed. Then, they were administered an intraperitoneal injection of 5 μL/g body mass LPS (5.0 mg/mL, Sigma, St. Louis, Mo., USA) and placed into triplicate tanks per treatment (26). Two fish per tank were anaesthetized and sampled at 0, 6, and 12 h after injection (n = 6 at each sampling time).

### 2.3 Sample collection

Blood was drawn from a vein using syringes rinsed with heparin sodium (0.2%) at 0, 6, and 12 h after LPS injection. The blood samples of six fish per tank were centrifuged at 3000 rpm for 15 min at 4°C to obtain the plasma, and the plasma was kept at −80°C. Parts of the liver were fixed in paraformaldehyde (4%) for histopathological evaluation or in 2.5% glutaraldehyde solution for ultrastructural observation. The remaining portions of the samples were frozen in liquid nitrogen and kept at −80°C.

### 2.4 Biochemical analysis

The levels of alanine aminotransferase (ALT, C009-2-1), aspartate aminotransferase (AST, C010-2-1), and lactate dehydrogenase (LDH, A020-2-2) in the serum, the activity of superoxide dismutase (SOD, A001-3-2), and the content of antioxidant capacity (T-AOC, A015-2-1), malondialdehyde (MDA, A003-1-2), lipid hydroperoxide (LPO, A106-1-3), and reactive oxygen species (ROS, 69-86537) in the liver were determined using commercial kits (Nanjing Jiancheng Bioengineering Institute, China). Complement C3 (C3, H186-1-2) and Complement C4 (C4, H186-2-2) were measured with ELISA kits (Nanjing Jiancheng Bioengineering Institute, China).

### 2.5 Histopathology

Hematoxylin and eosin (H&E) staining was carried out to evaluate liver injury. The paraffin-embedded liver tissues were cut into sections and stained with H&E, and the stained liver sections were examined under a microscope. We also examined hepatocyte apoptosis in the liver by terminal deoxynucleotidyl transferase-mediated dUTP nick-end labeling (TUNEL) and the ultrastructure of the liver *via* transmission electron microscopy (TEM), as described in a previous study (27).

### 2.6 Quantitative real-time polymerase chain reaction

The gene transcription levels related to oxidative stress, inflammation, mitochondrial dynamics, mitochondrial biogenesis, and mitophagy in liver samples from different treatment groups were analyzed. Total RNA was extracted with TRIzol reagent (Invitrogen, Carlsbad, USA) and then reverse transcribed to cDNA with M-MLV First-Strand Transcriptase (Invitrogen, Shanghai, China). Quantitative RT-PCR was performed on a LightCycle 480 II (Roche Diagnostics, Switzerland) instrument with SYBR Green Real-time PCR Master Mix (Roche Diagnostics, Switzerland). Relevant primers are listed in **Table 2**. Relative quantification of gene expression was performed as described by Pfaffl (28).

**Table 2:** Sequences of primers used for quantitative real-time PCR analysis in gibel carp.

Gene name	Sense and antisense primer (5’-3’)	Gene bank accession No.	Product length (bp)
β-actin	TTGAGCAGGAGATGGGAACCG	AB039726.2	115
	AGAGCCTCAGGGCAACGGAAA		
Nuclear factor [erythroid-derived 2]-like 2 (*nrf2*)	CCCTTCACCAAAGACAAGCA	MG759384	128
	TTGAAGTCATCCACAGGCAG		
Kelch-like ECH-associated protein-1 (*keap1*)	CTCACCCCCAACTTCCTGCAG	MG759382	150
	GATGAGCTGCGGCACCTTGGG		
NADPH quinine oxidoreductase-1 (*nqo-1*)	AGCAACAGAGACAACGGCAC	XM_026268231.1	176
	GTGTGCACCAGTACAGAGGAG		
Heme oxygenase-1 (*ho-1*)	GACAGGAGCATCTACCCACAG	KC758864.1	113
	GTGGCTGCTTTTATCTGCTCG		
Glutathione peroxidase (*gpx*)	GCCCACCCTCTGTTTGTGTT	DQ983598.1	244
	CAGGTTTATTTCGCCCTCTTC		
Superoxide dismutase (*sod*)	GTCCGCACTACAACCCTCAT	JQ776518.1	134
	GGTCACCATTTTATCCACAA		
Catalase (*cat*)	CTCCAACGGCAACTTCCCAT	JX477239.1	102
	CACACCTTAGTCAAATCAAA		
Nuclear factor-κB (*nfκb*)	CCTGCAAACACAGAACAACCC	KP125492.1	160
	GTCGTAGATGGGCTGAGACAC		
Tumor necrosis factor α (*tnf-α*)	TTGAGCAGGAGATGGGAACCG	XM_026282152.1	115
	AGAGCCTCAGGGCAACGGAAA		
Interleukin-6 (*il-6*)	TGTTCTCAGGGCATTCGCTT	XM_026289280.1	161
	GGAGTTGTAGTGCCCTTGGT		
Interleukin-1β (*il-1β*)	TTTGTGAAGATGCGCTGCTC	AB757758.1	133
	CCAATCTCGACCTTCCTGGTG		
Interleukin-2 (*il-2*)	GACCACAAAGGTAGACCCATCC	MN338056	212
	GAGGTTTGTGCGGAATGGAC		
Interleukin-12 (*il-12*)	CTTCAGAAGCAGCTTTGTTGTTG	LN592213.1	77
	CAGTTTTTGAGAGCTCACCAATATC		
Transforming growth factor β (*tgf-β*)	GGGTGGAGAGTTTATTACTGGCA	EU086521.1	186
	ACTCCTCTTCCTCGTCTACCTC		
Interleukin-4 (*il-4*)	TTGGCTCACTTTGCTTGAACTC	NW_020525339.1	284
	ATCACCCAATGTCTGTCTGTCC		
Interleukin-10 (*il-10*)	GTTGCTCATTTGTGGAGGGC	HQ259106.1	203
	AGCTGTTGGCAGAATGGTGT		
Dynamin-related protein 1 (*drp1*)	GGACCCAAAGCCAAATCAGAC	XM_026274285.1	228
	GGTACTTTGCATCTTGGTCGG		
Mitochondrial fission 1 (*fis1*)	CGTATTCAAAGGTCGTGTC	NC_039249.1	176
	GTTAGTCTGTGGTTATCGTCA		
Mitochondrial fission factor (*mff*)	TACGGCGATCTAAGTGCAAGC	NC_039263.1	262
	TGGTCGGTGTGAAGCTCAAG		
Mitofusin 1 (*mfn1*)	TAGTCGATATTGGTAGGCTTTG	NC_039269.1	214
	AGGGTCTTAAATCGTTCAGTTA		
Mitofusin 2 (*mfn2*)	CTCGGGATGCTTGATTCGCTT	XM_026270575.1	274
	GGTATTGGGTCGCGGGAAAA		
Optic atrophy 1 (*opa1*)	TTGTTAGCAGTGTAGCGGTGA	NC_039248.1	155
	GACTCCGAAGTGACAGAGTGA		
Nuclear respiratory factor 1 (*nrf1*)	ACGCTTACTGGATGAAGCTGAA	NC_039271.1	259
	GTGCTTAGTGGTTGAGGCAAAG		
Mitochondrial transcription factor A (*tfam*)	TGTAATGTATGCTGTCAGGGGT	NC_039279.1	104
	CAGTGCTTTGCAATTGTAGGTG		
Parkin RBR E3 ubiquitin-protein ligase (*parkin*)	TCCTGGGAGATGAGCAGTATGA	EU169139.1	190
	TTACACTCCCTGCCAAACACAA		
PTEN-induced kinase 1 (*pink1*)	TTTGAAGTGATGGCAGTTTATG	XM_026199593.1	186
	TGCATTCATTTGCCTACTTTTT		
Optineurin (*optn*)	AAAGGCTGAGTCGGAGGAAC	XM_026226962.1	122
	CCAACTCGGCAATTCTCCCA		
BCL2 interacting protein 3 (*bnip3*)	CATTTCCTTTCACTGCCTCGC	XM_042735968.1	93
	GATAGCCCTGGAATGGACTGG		
Microtubule-associated proteins 1A/1B light chain 3B (*lc3b*)	CTACGAGCGCGAGAGAGATG	XM_026238789.1	81
	TGAGGACACGCAGTTCCAAA		
P62 (*p62*)	AGGACGACTGCAACAAGGAG	XM_026279678.1	100
	CCGACGAAAACATCGAGCAC		

### 2.7 Western blot analysis

The weighed liver samples were homogenized in RIPA lysis buffer (Beyotime Biotechnology, Shanghai, China) and centrifuged to obtain the supernatant, and the protein concentration was measured using a protein analysis kit (Beyotime Biotechnology, Shanghai, China). Protein preparations (20 μg) were separated by electrophoresis using 6%, 8%, and 12% SDS-polyacrylamide gels (SDS-PAGE) and then transferred onto polyvinylidene fluoride (PVDF) membranes. The membranes were blocked with 5% skim milk in 0.1% Tween-20 (TBST) buffer for 1 h and further incubated with specific primary antibodies GAPDH (#5174, Cell signaling, Danvers, MA, USA), SIRT1 (A17307, Abclonal, Wuhan, China), PGC-1α (A12348, Abclonal, Wuhan, China), P62 (A11247, Abclonal, Wuhan, China), and LC3A/B-I/II (4108S, Cell signaling, Danvers, MA, USA). The membranes were then incubated with the secondary antibody Goat Anti-Rabbit IgG H&L (HRP) (1:5000, ab205718; Abcam), and each membrane was ultimately scanned using a CCD camera (Chemidoc MP Imaging System, BioRad) to evaluate the intensity of bands.

### 2.8 Immunofluorescence analysis

The liver samples were made into paraffin sections and dewaxed by successively soaking in xylene (two times, 15 min each time), absolute ethanol (two times, 5 min each time), 85% alcohol (5 min), and 75% alcohol (5 min). The sections were placed in citric acid repair solution (pH = 6.0) for antigen repair and then washed three times with PBS (pH = 7.4) for 5 min each time. Endogenous peroxidase was blocked with hydrogen peroxide solution (3%), and the serum was blocked with BSA (3%). Then, the sections were incubated with the first antibody to SIRT1, PGC-1α, P62, or LC3B overnight at 4°C. P62 and LC3B were subjected to co-localization analysis. After PBST cleaning, the sample was incubated at room temperature for 50 min with HRP-labeled goat anti rabbit (SeraCare, 1:400). TSA (Tyramine-488, 1/200) was added to the liver samples, and the nuclei were stained with DAPI. Finally, the sections were observed under a fluorescence microscope.

### 2.9 Statistical analysis

Statistical analyses within the same group were conducted using one-way ANOVA, and comparisons at the same time point were analyzed with independent sample t-tests using SPSS version 26.0 (SPSS Inc., Chicago, USA). Comparisons between experimental groups were considered to be significantly different at *P* < 0.05 and extremely significant at *P* < 0.01. Results were presented as mean ± SEM.

## 3 Results

### 3.1 Plasma metabolites

**Figure 1:**
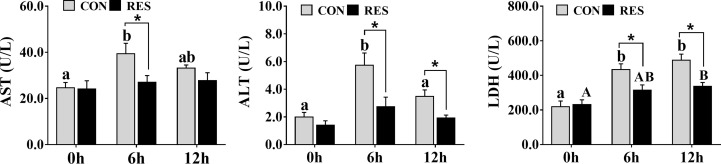
Effect of dietary RES on plasma AST, ALT, and LDH activities in gibel carp at 6 h and 12 h after LPS injection. Data are mean ± SEM (n = 6). The lowercase letters represent significant differences among diverse LPS durations in the CON group (*P* < 0.05); the uppercase letters represent significant differences among diverse LPS durations in the RES group (*P* < 0.05). * (*P* < 0.05) represent significant differences between the CON and RES groups.

The levels of AST, ALT, and LDH in the plasma are shown in **Figure 1**. The AST, ALT, and LDH levels were significantly increased at both 6 h and 12 h after LPS injection. It is worth noting that RES significantly reduced the levels of AST, ALT, and LDH in the plasma, except for the AST level at 6 h after injection.

### 3.2 Tissue injury in liver and hepatocyte apoptosis

**Figure 2:**
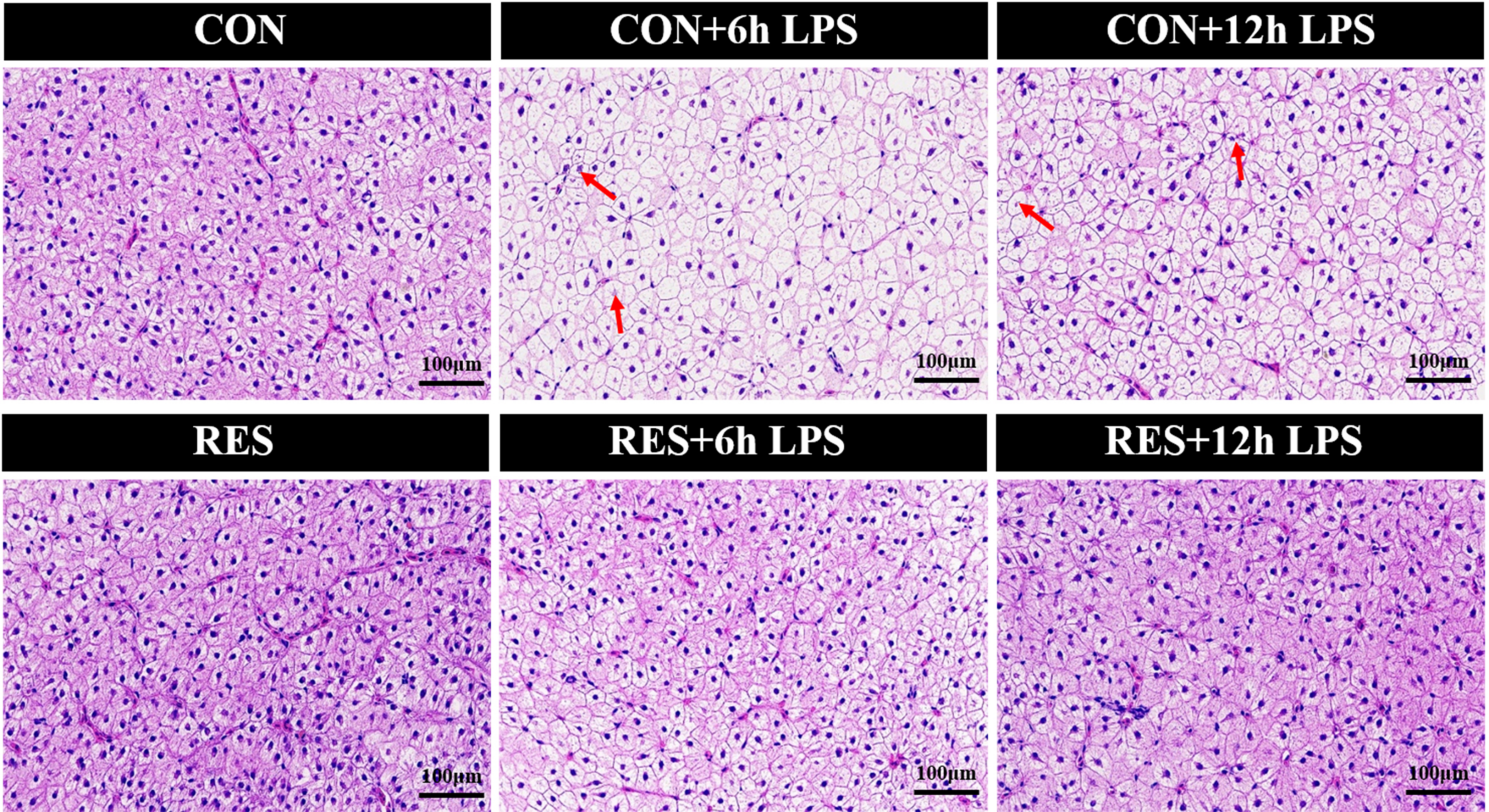
Effect of dietary RES on H&E staining of liver tissue in gibel carp at 6 h and 12 h after LPS injection.

As shown in **Figure 2**, the liver tissue structure of gibel carp in the CON group and RES group was basically normal. However, at 6 h and 12 h after LPS injection, the hepatocytes of the fish in the CON group showed abnormalities, with mild swelling of hepatocytes in the field of vision, vacuolation of cytoplasm, necrosis of some hepatocytes, and disappearance or fragmentation of some nuclei (**Figure 2**). Compared to the CON group, the liver injury of fish in the RES group was alleviated, being characterized by a complete hepatocyte structure, uniform size of hepatic sinuses, and no inflammatory cell infiltration.

**Figure 3:**
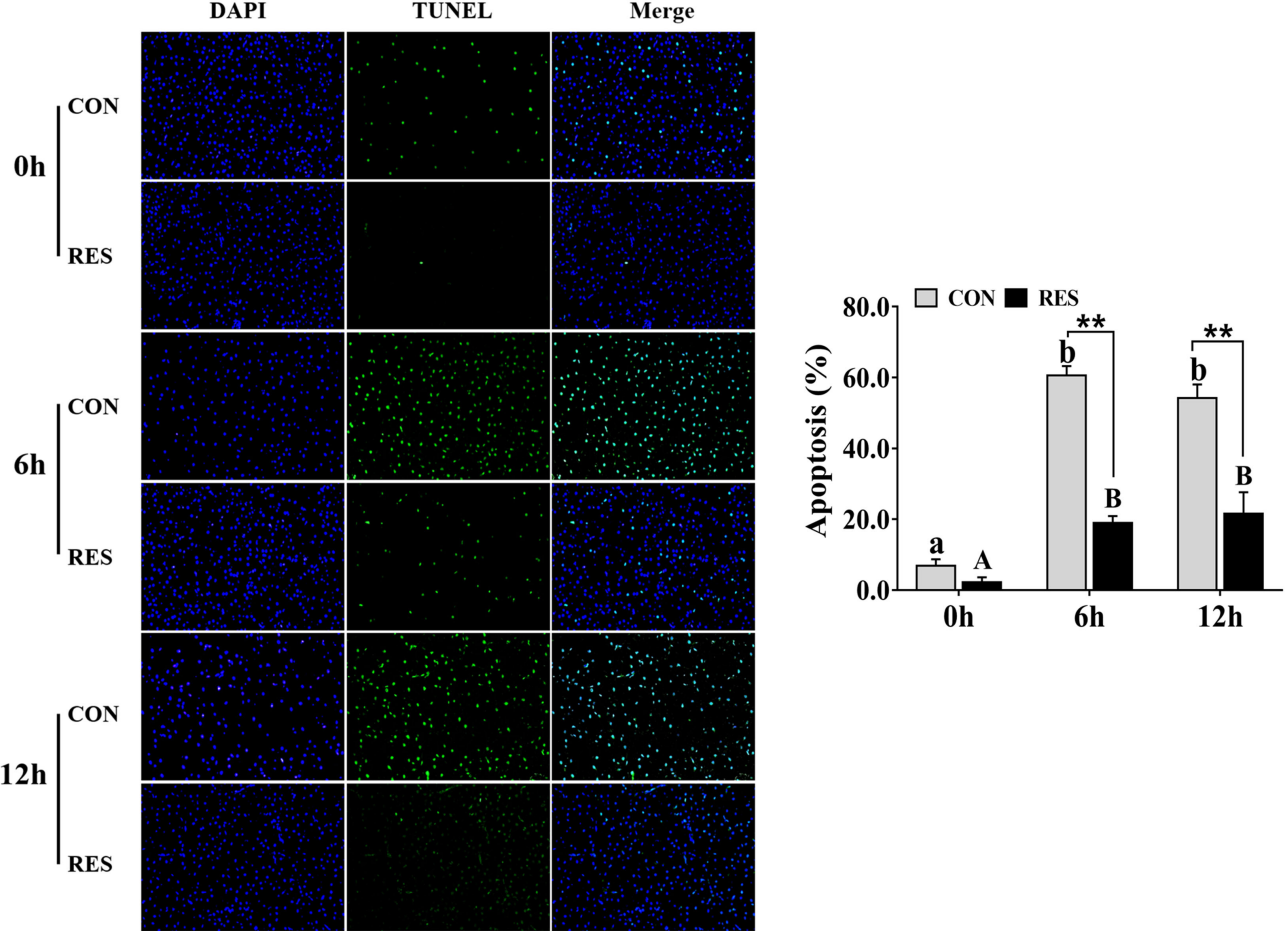
Effect of dietary RES on hepatocyte apoptosis in gibel carp at 6 h and 12 h after LPS injection. Data are mean ± SEM (n = 6). The lowercase letters represent significant differences among diverse LPS durations in the CON group (*P* < 0.05); the uppercase letters represent significant differences among diverse LPS durations in the RES group (*P* < 0.05). ** (*P* < 0.01) represents significant differences between the CON and RES groups.

In addition, the apoptosis signal of hepatocytes in gibel carp was significantly increased after LPS administration (**Figure 3**). Nevertheless, RES significantly reduced the rate of apoptosis in hepatocytes after LPS stimulation.

### 3.3 Oxidative stress in the liver and expression of antioxidant-related genes in hepatocytes

**Figure 4:**
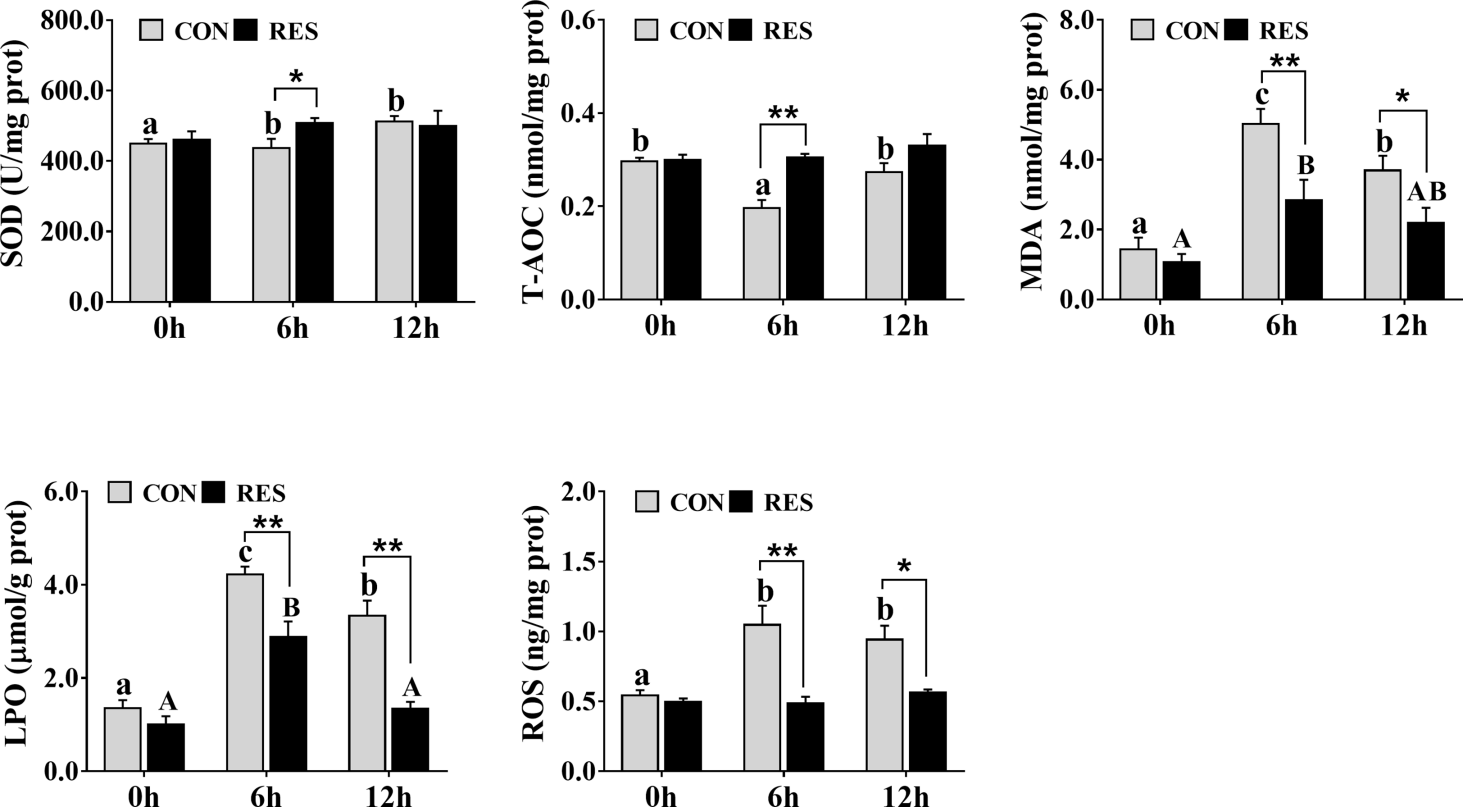
Effect of dietary RES on LPS-induced oxidative stress in the liver of gibel carp at 6 h and 12 h after LPS injection. Data are mean ± SEM (n = 6). The lowercase letters represent significant differences among diverse LPS durations in the CON group (*P* < 0.05); the uppercase letters represent significant differences among diverse LPS durations in the RES group (*P* < 0.05). *(*P* < 0.05) and **(*P* < 0.01) represent significant differences between the CON and RES groups.

As shown in **Figure 4**, SOD activity was increased at both 6 h and 12 h after LPS injection in the CON group, but T-AOC was decreased at 6 h. However, compared to the CON group, there was a significant increase in the RES group at 6 h after LPS injection. The MDA and LPO contents in the liver tissue of gibel carp of the CON and RES groups were increased significantly at 6 h and 12 h after LPS injection (**Figure 4**), and the difference was statistically significant between the CON and RES groups. At 6 h and 12 h, both MDA and LPO contents in the RES group were dramatically decreased. Similar changes were also found in ROS.

**Figure 5:**
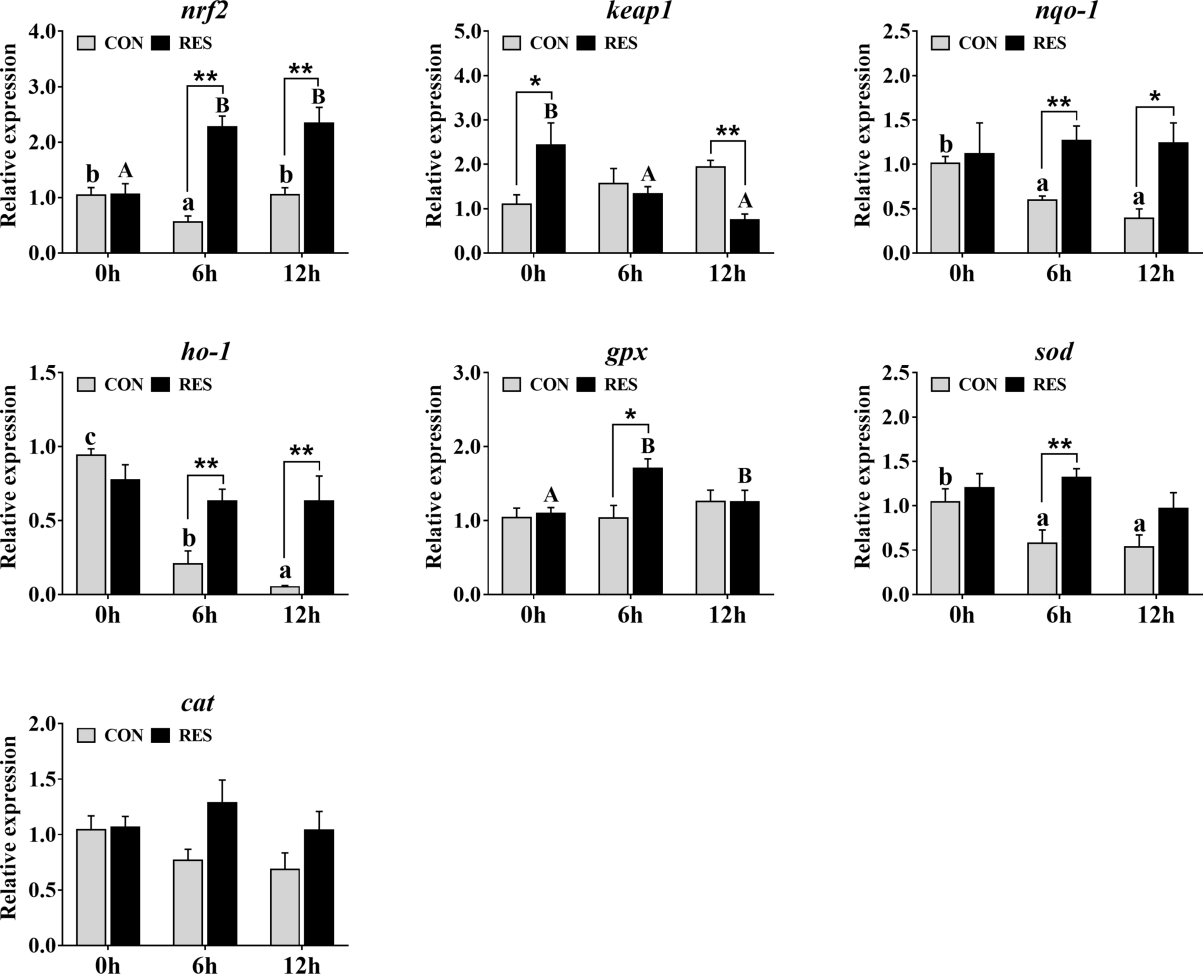
Effect of dietary RES on mRNA expression levels of antioxidant-related genes in the liver of gibel carp at 6 h and 12 h after LPS injection. The lowercase letters represent the significant differences among diverse LPS durations in the CON group (*P* < 0.05); the uppercase letters represent the significant differences among diverse LPS durations in the RES group (*P* < 0.05). *(*P* < 0.05) and **(*P* < 0.01) represent significant differences between the CON and RES groups. *Nrf2*: nuclear factor [erythroid-derived 2]-like 2; *keap1*: kelch-like ECH-associated protein-1; *nqo-1*, NADPH quinine oxidoreductase-1; *ho-1*, heme oxygenase-1; *gpx*, glutathione peroxidase; *sod*, superoxide dismutase; *cat*, catalase.

The transcriptional levels of antioxidant-related genes in the liver of gibel carp at 0, 6, and 12 h after LPS injection are shown in **Figure 5**. At 6 h after LPS injection, the mRNA expression levels of *nrf2*, *nqo-1*, *ho-1*, and *sod* in the CON group were significantly decreased. At 6 h and 12 h after LPS injection, the transcript levels of *nrf2* and *gpx* in the RES group were increased, while that of *keap1* was decreased. Compared with the CON group, RES significantly upregulated the expression levels of *nrf2*, *nqo-1*, and *ho-1* at 6 and 12 h after LPS injection and that of *gpx* and *sod* at 6 h after LPS injection. In contrast, *keap1* was significantly downregulated in the RES group at 12 h after LPS injection. No significant differences in *cat* were observed.

### 3.4 Immune response in plasma and the expression of inflammation-related genes in hepatocytes

**Figure 6:**
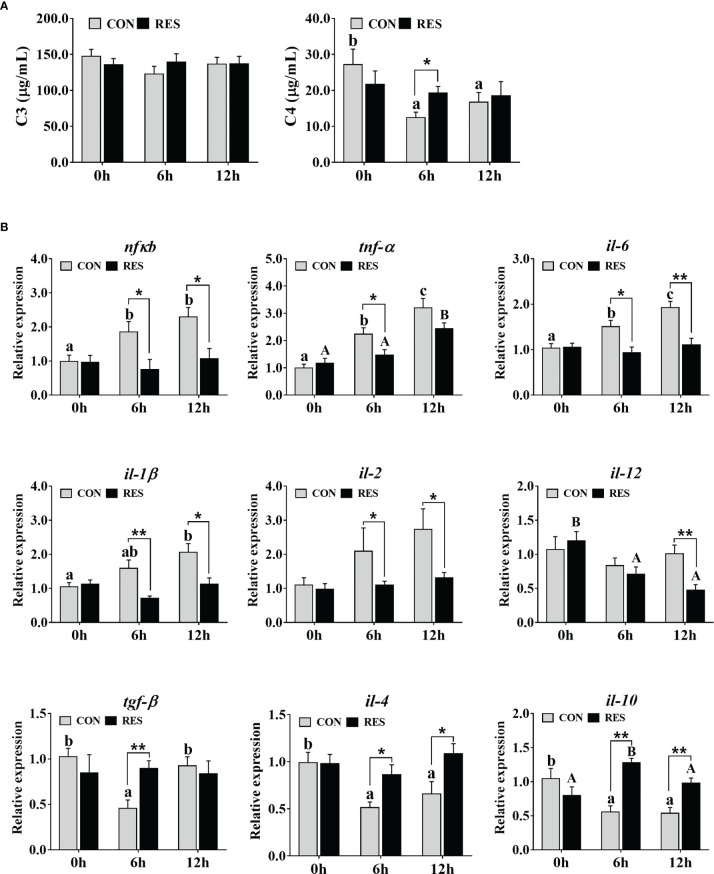
Effect of dietary RES on immune reaction in plasma **(A)** and mRNA expression levels of inflammation-related genes in the liver **(B)** of gibel carp at 6 h and 12 h after LPS injection. The lowercase letters represent significant differences among diverse LPS durations in the CON group (*P* < 0.05); the uppercase letters represent significant differences among diverse LPS durations in the RES group (*P* < 0.05). *(*P* < 0.05) and **(*P* < 0.01) represent significant differences between the CON and RES groups. *Nfκb*, nuclear factor-κB; *tnf-α*, tumor necrosis factor α; *il-6*, interleukin-6; *il-1β*, interleukin-1β; *il-2*, interleukin-2; *il-12*, interleukin-12; *tgf-β*, transforming growth factor β; *il-4*, interleukin-4; *il-10*, interleukin-10.

As shown in **Figure 6A**, C4 was decreased in the plasma of gibel carp at 6 h and 12 h after LPS injection but was induced by RES. No significant difference was observed in C3.

The transcript levels of inflammation-related genes in the liver of gibel carp at 0, 6, and 12 h after LPS injection are shown in **Figure 6B**. LPS enhanced the expression of the pro-inflammatory genes *nfκb*, *tnf-α*, *il-6*, and *il-1β* but inhibited the expression of the anti-inflammatory genes *tgf-β*, *il-4*, and *il-10* in the CON group. Higher expression levels of *tnf-α* and *il-10* but lower expression levels of *il-12* were found in fish fed the RES diet. Compared to the CON group, the mRNA levels of *nfκb*, *tnf-α*, *il-6*, *il-1β*, *il-2*, and *il-12* were dramatically downregulated, while that of *tgf-β*, *il-4*, and *il-10* was upregulated in the RES group.

### 3.5 Mitochondrial fission and fusion related genes in hepatocytes

**Figure 7:**
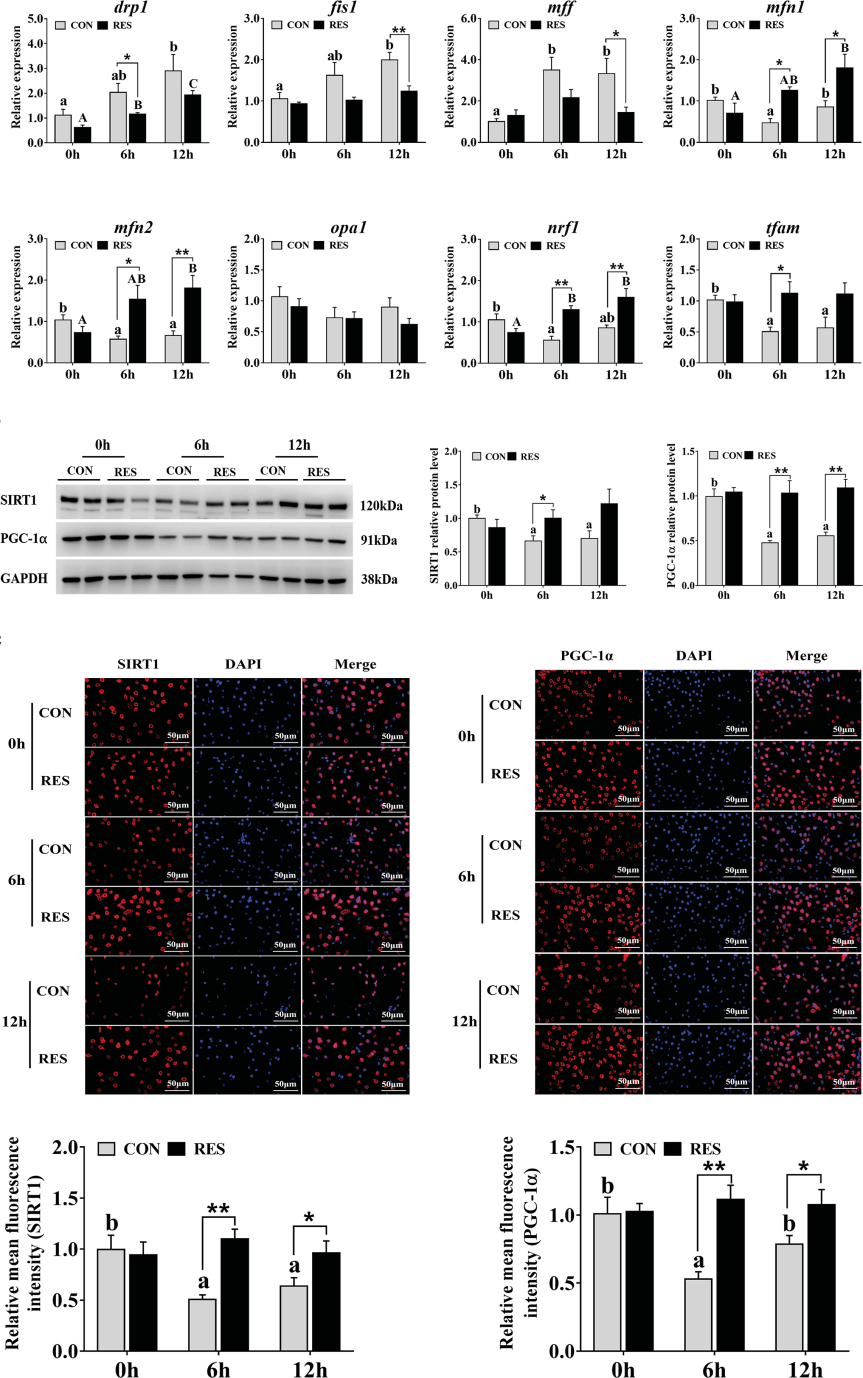
Effect of dietary RES on mitochondrial dynamics and mitochondrial biogenesis in the liver of gibel carp at 6 h and 12 h after LPS injection. **(A)** Mitochondrial dynamics related mRNA levels were determined by qRT-PCR. **(B)** Western blots for SIRT1 and PGC-1α and quantification results. **(C)** Immunofluorescent analysis of SIRT1 and PGC-1α stained with antibody (red) or DAPI (blue) and relative fluorescence intensity. Bars = 50 µm. Data are mean ± SEM (n = 6). The lowercase letters represent significant differences among diverse LPS durations in the CON group (*P* < 0.05); the uppercase letters represent significant differences among diverse LPS durations in the RES group (*P* < 0.05). *(*P* < 0.05) and **(*P* < 0.01) represent significant differences between the CON and RES groups. *Drp1*, dynamin-related protein 1; *fis1*, mitochondrial fission 1; *mff*, mitochondrial fission factor; *mfn1*, mitofusin 1; *mfn2*, mitofusin 2; *opa1*, optic atrophy 1; *nrf1*, nuclear respiratory factor 1; *tfam*, mitochondrial transcription factor A.

The mRNA levels of mitochondrial fission and fusion related genes in the liver are shown in **Figure 7A**. The *drp1*, *fis1*, and *mff* expression levels were upregulated, and the transcript levels of *mfn1*, *mfn2*, *nrf1*, and *tfam* were downregulated by LPS. In addition, the mRNA expression levels of *mfn1*, *mfn2*, *nrf1*, and *tfam* were significantly enhanced by RES after LPS injection. Compared with the CON group, decreased *drp1*, *fis1*, and *mff* expression levels were found in the RES group.

### 3.6 Mitochondrial biogenesis related SIRT1-PGC1α pathway

The expression of key proteins involved in the SIRT1-PGC1α signaling pathway in the liver of gibel carp injected with LPS is shown in **Figure 7B**. Both SIRT1 and PGC-1α relative protein levels were significantly inhibited in the CON group, while no change was found in the RES group. However, at 6 h after LPS injection, the levels of SIRT1 protein in the RES group were significantly enhanced compared to that in the CON group. PGC-1α levels were also increased at both 6 h and 12 h in the RES group. Similar changes were found in the results of immunofluorescence staining (**Figure 7C**).

### 3.7 Mitophagy-related SIRT1-P62/LC3B pathway

**Figure 8:**
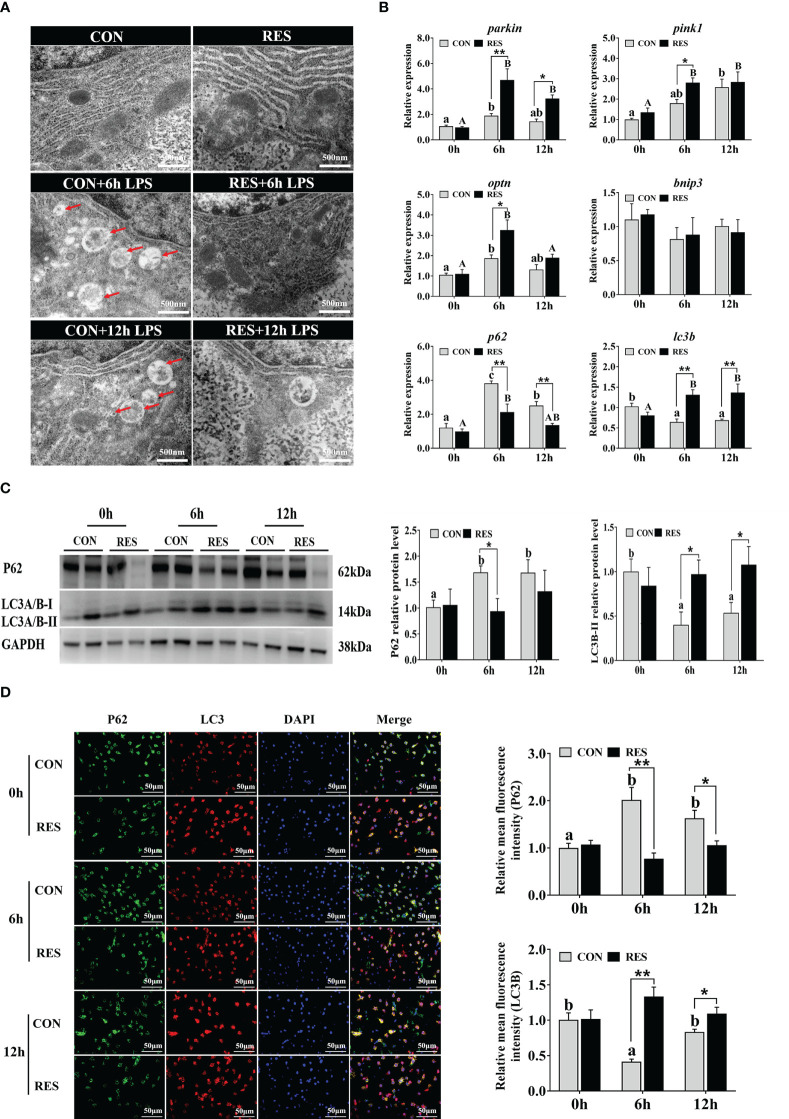
Effect of dietary RES on mitochondrial autophagy in the liver of gibel carp at 6 h and 12 h after LPS injection. **(A)** Ultrastructural changes of hepatocytes under TEM. Scale bar = 500 nm. Red arrows represent the autophagosome. **(B)** Mitophagy-related mRNA levels were determined with qRT-PCR. **(C)** Western blots for P62 and LC3B and quantification results. **(D)** Co-localization analysis of P62 (green) and LC3 (red) in the hepatocytes of gibel carp and the relative fluorescence intensity. DAPI staining is shown in blue. Bars = 50 µm. Data are mean ± SEM (n = 6). The lowercase letters represent significant differences among diverse LPS durations in the CON group (*P* < 0.05); the uppercase letters represent significant differences among diverse LPS durations in the RES group (*P* < 0.05). *(*P* < 0.05) and **(*P* < 0.01) represent significant differences between the CON and RES groups. *Parkin*, parkin RBR E3 ubiquitin-protein ligase; *pink1*, PTEN-induced kinase 1; *optn*, optineurin; *bnip3*, BCL2 interacting protein 3; *lc3b*, microtubule-associated proteins 1A/1B light chain 3B; *p62*, P62.

As shown in **Figure 8A**, LPS increased the number of autophagosomes only in the CON group. The mRNA levels of *parkin*, *pink1*, *optn*, and *p62* were significantly upregulated by LPS (**Figure 8B**). Conversely, *lc3b* levels were inhibited after LPS administration. The expression levels of *parkin*, *pink1*, *optn*, and *lc3b* were enhanced by RES, whereas *p62* expression was inhibited. No significant difference was found in *bnip3* gene expression. Consistent results were found in protein expression levels of *p62* and *lc3b* detected by Western blotting (**Figure 8C**) and fluorescence intensity detected by immunofluorescence staining (**Figure 8D**).

## 4 Discussion

LPS can activate the immune response in fish to trigger liver injury (6). Therefore, in this study, LPS was administered to gibel carp after 8 weeks of breeding, and samples were taken at 6 h and 12 h post-injection. The results showed that LPS induced significant increases in the plasma levels of AST, ALT, and LDH in gibel carp at 6 h post-injection, while by 12 h, the levels of AST and ALT were decreased compared to those at 6 h. ALT and AST are released into the plasma as markers of liver injury; elevated levels of LDH, which catalyzes the dehydrogenation of lactate to pyruvate, indicate liver dysfunction (29). Thus, the results suggested that liver injury was induced by LPS injection in the gibel carp and that the damage was time-dependent. This may have been due to the gradual metabolism of LPS by gibel carp and the cells themselves responding to external stimuli. Similarly, temporal differences were observed in mice, with plasma ALT and AST levels increasing and then decreasing during 12–72 h after LPS treatment (30). RES is a plant antitoxin polyphenol that attenuates LPS-induced hepatotoxicity in mice through changes in iron shuttle protein (31). In addition, RES has also been shown to alleviate hepatic immunotoxicity and liver injury in H_2_O_2_-treated tilapia (32). In this study, RES significantly reduced plasma AST, ALT, and LDH levels and alleviated liver injury in gibel carp after LPS injection.

After 6 h and 12 h of LPS treatment, liver HE staining showed mild swelling and even necrosis of the hepatocytes of the fish liver, accompanied by loss or fragmentation of nuclei, with significantly increased apoptosis. This suggested that LPS induced hepatocyte injury and apoptosis. Similarly, zebrafish hepatocytes were sparsely connected and showed nuclear atrophy and lysis after LPS treatment (8). In mice, TUNEL-positive staining of hepatocytes was significantly increased after LPS treatment (33). However, the liver tissues of gibel carp were basically restored to normal with intact and well-defined hepatocytes, and the number of apoptotic hepatocytes under TUNEL staining was significantly reduced in the RES group after LPS administration. Therefore, RES could alleviate the liver injury and significantly reduce the apoptosis of hepatocytes in gibel carp after LPS injection.

Oxidative stress is an imbalance between increased ROS and reduced activity of antioxidant mechanisms during physiological processes, resulting in severe damage to macromolecules (34). ROS react with lipids to induce lipid peroxidation, which directly responds to free radical damage. MDA, a metabolic end product of lipid peroxidation, is a reliable indicator of oxidative stress and inflammatory responses, and its inhibition is an effective way to combat oxidative stress (35). In this study, the MDA, LPO, and ROS levels in the liver of gibel carp were increased significantly at 6 h and 12 h after LPS injection and were decreased significantly after RES treatment, suggesting that RES can effectively alleviate LPS-induced oxidative stress. Consistent with this study, RES alleviated muscle oxidative damage in southern flounder (21). Temporally, the MDA and LPO levels in the liver were significantly lower at 12 h than at 6 h after LPS injection, indicating a reduction in lipid peroxidation. SOD is an important antioxidant enzyme in the endogenous antioxidant system, catalyzing the conversion of superoxide to oxygen and hydrogen peroxide and resisting oxidative damage (36). It was previously reported that LPS treatment reduced the antioxidant capacity of rats (31). In our study, liver SOD activity was significantly increased at 6 and 12 h after LPS injection, but T-AOC levels were remarkably reduced at 6 h. However, RES significantly increased SOD activity and T-AOC levels at 6 h compared to that in the CON group. Thus, our data indicated that LPS impaired the antioxidant system of gibel carp but reduced lipid peroxidation by enhancing antioxidant enzyme activity. Furthermore, RES could mitigate extra ROS by enhancing antioxidant capacity.

Nrf2 is critical in the endogenous antioxidant defense system, and its activation and inhibition are correlated with the degree of oxidative stress (37). In mice with acute lung injury, Galectin-1 significantly upregulated LPS-induced low expression of Nrf2 as well as the downstream antioxidant protective proteins HO-1 and NQO1 (38). In this study, the mRNA expression of *nrf2* and downstream *nqo-1*, *ho-1*, and *sod* in gibel carp was significantly downregulated at 6 h and 12 h after LPS injection, which may be the result of Nrf2 inactivation. Previous study has shown that RES attenuated LPS-induced acute ileitis in ducks by mediating the phosphorylation of Nrf2 (39). Our results showed that RES treatment remarkably enhanced the gene expression of *nrf2*, *nqo-1*, *ho-1*, *gpx*, and *sod* compared with the CON group at 6 h after LPS injection. At 12 h after LPS injection, RES significantly enhanced the expression of *nrf2*, *nqo-1*, and *ho-1*. Thus, RES may protect against LPS-induced oxidative stress in gibel carp by upregulating the Nrf2 pathway, and its antioxidant effect was more pronounced at 6 h after LPS injection.

As key components of the immune response in fish, C3 and C4 play crucial roles in pathogen detection and clearance (40). In carp, LPS treatment significantly reduced the immunity of the fish (9). In this study, plasma C4 levels of gibel carp were significantly reduced after LPS injection. Compared with the CON group, RES significantly increased plasma C4 levels at 6 h. Numerous studies have shown that NF-κB is an important regulatory protein that is widely present in eukaryotic cells and is involved in the regulation of inflammatory responses in fish (6, 10, 41, 42). In grass carp, soybean β-globulin drove NF-κB P65 nuclear translocation and TOR phosphorylation to regulate inflammatory cytokines (41). In carp, the insecticide avermectin induced an inflammatory response by activating NF-κB pathways to release inflammatory factors that disrupted the blood-brain barrier structure (42). In addition, NF-κB has also been shown to regulate fish cytokines in the inflammatory response in gibel carp (6) and yellow catfish (10). Significant upregulation of *nfkb* and its downstream inflammatory factors *thf-α*, *il-6*, and *il-1β*, but significant downregulation of anti-inflammatory factors *tgf-β*, *il-4*, and *il-10* at 6 h and 12 h after LPS injection suggested that severe inflammation was induced. However, RES reversed the expression of these genes, especially at 6 h after LPS injection. Similarly, RES exerted its antioxidant properties by regulating the Nrf2 and TLR2-Myd88-NF-κB signaling pathways that significantly attenuated the liver immunotoxicity in H_2_O_2_-treated tilapia (32). Taken together, the results suggest that RES may alleviate LPS-induced inflammation in gibel carp by activating Nrf2 and concurrently inhibiting the NF-κB pathway.

Mitochondria are important sites of ROS production and are particularly susceptible to oxidative stress (43). It has been demonstrated that LPS caused mitochondrial dysfunction through oxidative stress and excessive mitochondrial fission and that this may be a potential mechanism of inducing sepsis (44). In this study, the expression levels of the mitochondrial division genes *drp1*, *fis1*, and *mff* were significantly upregulated, while the mitochondrial fusion genes *mfn1*, *mfn2*, *nrf1* and the mitochondrial transcription factor (*tfam*) were significantly downregulated at 6 h and 12 h after LPS injection, implying that LPS may stimulate a reduction in high-quality mitochondria, inhibit mitochondrial fusion, and promote mitochondrial division (45). PGC-1α is a major regulator of mitochondrial biosynthesis, whose activation is mediated by SIRT1-dependent deacetylation of NAD^+^, which activates and controls NRF1 and NRF2 and subsequent TFAM transcription (17). In this study, protein expression and antigen levels of SIRT1 and PGC-1α were significantly downregulated at 6 h and 12 h after LPS injection, demonstrating that LPS significantly interfered with the biological process of mitochondrial genesis in gibel carp. RES is a SIRT1 activator, and it enhanced the transcriptional regulation of SIRT1 to alleviate Cd-induced inhibition of PGC-1α and TFAM expression (46). Furthermore, RES increased the deacetylase activity of SIRT1 and reduced aberrant mitochondria in mice exposed to manganese (47). In gibel carp, RES significantly downregulated LPS-induced high expression of *drp1*, *fis1*, and *mff* and upregulated low expression of *mfn1*, *mfn2*, *nrf1*, and *tfam* by activating SIRT1 and PGC-1α. In addition, bisdemethoxycurcumin activated the antioxidant defense system and regulated mitochondrial biogenesis by upregulating PGC-1α and promoting the nuclear localization of Nrf2, repairing oxidative damage caused by LPS injection and maintaining mitochondrial function in broilers (48). Thus, combined with the upregulation of Nrf2 by RES, RES may induce the antioxidant system and mitochondrial biogenesis by activating the SIRT1-PGC-1α and Nrf2-Keap1 pathways to alleviate mitochondrial dysfunction after LPS injection in gibel carp.

When mitochondria were disrupted, PINK1 mediated the maturation of autophagosomes and their fusion with lysosomes by activating and recruiting parkin (49). Mitochondrial autophagy acts as a protective mechanism to remove damaged mitochondria and reduce excessive release of ROS through autophagosome formation, transport to lysosomes, and lysosomal degradation (15). The presence of autophagosomes indicates that LPS-induced mitochondrial dysfunction was ameliorated (43). In this study, autophagosomes were induced in the hepatocytes of gibel carp at 6 h and 12 h after LPS injection compared to the CON group. Similarly, the number of autophagosomes was increased in LPS-induced depressed mice (50). The expression levels of mitophagy-related genes *parkin*, *pink1*, and *optn* were upregulated at 6 h and 12 h after LPS injection. However, these genes were further enhanced by RES, and no autophagosomes were observed in the RES group, indicating that RES inhibited mitophagy and the formation of autophagosomes by mediating the PINK/Parkin pathway induced by LPS in gibel carp.

During mitophagy, LC3 proteins are hydrolytically cleaved to form LC3-I and subsequently processed and modified by ubiquitin-like systems to form LC3-II that is localized to the autophagosomal membrane (51). In late mitophagy, p62 binds to ubiquitinated proteins and forms a complex with LC3 to target mitochondria in the autophagosome (49, 52). It has been reported that autophagy receptor p62 recruitment in brain-injured mice promoted Parkin-dependent mitophagy, accompanied by increased LC3-II levels (53). In both *in vivo* and *in vitro* models, berberine enhanced mitophagy by increasing the LC3-II/LC3-I ratio and PINK1/Parkin protein levels and decreasing P62 protein levels, thereby protecting against acute kidney injury in mice (54). In this study, the *p62* expression level was significantly enhanced at 6 h and 12 h after LPS injection, but *lc3b* was significantly reduced. Similar changes in the protein expression levels of P62 and LC3B-II/I were observed in the liver of gibel carp, confirming the occurrence of mitophagy to form autophagosomes induced by LPS. In contrast, opposite changes were found in the RES group, where the formation of LC3 complexes and the degradation of proteins after autophagy in the liver of gibel carp were promoted by RES.

## 5 Conclusions

We explored the effects of RES on oxidative stress, immunity, mitochondrial biogenesis, and mitophagy induced by LPS injection in gibel carp. The results demonstrated that a RES diet further reduced the stress in gibel carp by activating the Nrf2/Keap1 pathway and inhibiting the NF-κB pathway. RES alleviated the oxidative stress and inflammation by activating the Nrf2/Keap1 pathway and inhibiting the NF-κB pathway induced by LPS. Furthermore, RES enhanced mitochondrial autophagy and mitochondrial biogenesis to mitigate ROS accumulation by activating the SIRT1-PGC-1α and PINK/Parkin signaling pathways. Thus, RES regulated mitochondrial quality control to deal with oxidative stress and inflammation in LPS-injected gibel carp. Our results could provide a strategy for enhancing fish immunity in aquaculture.

## Data availability statement

The original contributions presented in the study are included in the article/supplementary material. Further inquiries can be directed to the corresponding author.

## Ethics statement

The animal study was reviewed and approved by the ethics committee of the Institute of Hydrobiology, Chinese Academy of Sciences.

## Author contributions

LW, and QC performed the experiments, analyzed data, and drafted the manuscript. BD, HG, and YW researched data and contributed to the discussion. DH, XZ, and HL conducted the investigation. YY participated in the methodology. SX performed the funding acquisition and reviewed the draft. JJ performed the conceptualization, supervision, and funding acquisition, edited and revised the paper. All authors contributed to the article and approved the submitted version.
